# Machine learning for understanding and predicting neurodevelopmental outcomes in premature infants: a systematic review

**DOI:** 10.1038/s41390-022-02120-w

**Published:** 2022-05-31

**Authors:** Stephanie Baker, Yogavijayan Kandasamy

**Affiliations:** 1 College of Science and Engineering, James Cook University, Cairns, QLD 4878 Australia; 2 Department of Neonatology, Townsville Hospital and Health Service, Townsville, QLD 4810 Australia; 3 College of Medicine and Dentistry, James Cook University, Townsville, QLD 4810 Australia

## Abstract

**Background:**

Machine learning has been attracting increasing attention for use in healthcare applications, including neonatal medicine. One application for this tool is in understanding and predicting neurodevelopmental outcomes in preterm infants. In this study, we have carried out a systematic review to identify findings and challenges to date.

**Methods:**

This systematic review was conducted in accordance with the Preferred Reporting Items for Systematic Reviews and Meta-Analysis guidelines. Four databases were searched in February 2022, with articles then screened in a non-blinded manner by two authors.

**Results:**

The literature search returned 278 studies, with 11 meeting the eligibility criteria for inclusion. Convolutional neural networks were the most common machine learning approach, with most studies seeking to predict neurodevelopmental outcomes from images and connectomes describing brain structure and function. Studies to date also sought to identify features predictive of outcomes; however, results varied greatly.

**Conclusions:**

Initial studies in this field have achieved promising results; however, many machine learning techniques remain to be explored, and the consensus is yet to be reached on which clinical and brain features are most predictive of neurodevelopmental outcomes.

**Impact:**

- This systematic review looks at the question of whether machine learning can be used to predict and understand neurodevelopmental outcomes in preterm infants.
- Our review finds that promising initial works have been conducted in this field, but many challenges and opportunities remain.
- Quality assessment of relevant articles is conducted using the Newcastle–Ottawa Scale.
- This work identifies challenges that remain and suggests several key directions for future research.
- To the best of the authors’ knowledge, this is the first systematic review to explore this topic.

## Introduction

Premature birth is a well-known cause of long-term neurodevelopmental difficulties and disabilities.^1^ Currently, 15 million infants are born prematurely at less than 37 weeks gestational age annually, and this rate continues to increase worldwide.^2^ Improvements in neonatal healthcare are driving an increase in survival rates amongst extremely premature infants;^3–7^ however, challenges remain in improving the neurodevelopmental outcomes of preterm infants.^8^

Fortunately, early intervention programs for preterm infants have a positive influence on neurodevelopmental outcomes during infancy, with cognitive benefits persisting into preschool age.^9,10^ Early intervention requires early identification of at-risk infants, which remains a significant challenge for clinicians.

One tool that offers significant promise for identifying infants at risk is machine learning (ML). To date, ML models have been developed for the prediction of many outcomes in premature infants, including mortality,^11^ sepsis,^12^ and retinopathy of prematurity.^13,14^ However, relatively few have investigated neurodevelopment. To the best of our knowledge, no systematic review on using ML to predict neurodevelopmental outcomes in premature infants has been conducted. This review aims to fill that gap in the literature, while identifying strengths and weaknesses of existing approaches and identifying several future research directions.

## Methods

This study presents a systematic review on the prediction of neurodevelopmental outcomes in preterm infants using ML. The review was completed in accordance with the Preferred Reporting Items for Systematic Reviews and Meta-Analysis guidelines.^15^

### Data sources and searching

We conducted a systematic search of four electronic databases: PubMed, OVID Medline, CINAHL, and SCOPUS. The search strategy used for each database consisted of the following keywords: ((machine AND learning) OR (deep AND learning)) AND (neonatal OR neonate OR preterm OR premature OR baby OR infant) AND (language OR speech OR neurodevelopment). The searches focused on articles that contained these keywords in the title, abstract, or keywords. Gray literature was not considered by this systematic review.

### Inclusion and exclusion criteria

This review focuses on the question of whether ML techniques can be used to predict neurodevelopmental outcomes in preterm infants. Studies were deemed eligible if they were peer-reviewed and published in English between January 2010 and February 2022 (including early-access and pre-print articles). Studies predating January 2010 were excluded as ML is a rapidly evolving field and thus only recent studies are relevant.

As this systematic review focuses on outcomes in premature infants, only studies that included a cohort of preterm infants with postmenstrual ages ≤37 weeks are included. Any studies that did not include a cohort of preterm infants were excluded. Studies that considered solely maternal or fetal health factors were also excluded. Animal studies were not relevant to this study and thus were excluded.

Only articles focusing on general neurodevelopmental outcomes were included; thus articles that focused on predicting or understanding physical growth or specific conditions (such as cerebral palsy and Angelman syndrome) were excluded.

### Data extraction and article selection

The final search was conducted in February 2022. All results were imported to Mendeley, where duplicates were automatically identified and then manually confirmed for removal. The titles and abstracts of the identified articles were independently screened in a non-blinded manner by the two authors. The full text was then examined for each of the remaining studies to enable a final decision on eligibility for inclusion. Disagreements were resolved through discussion, with consensus reached for each study.

## Results

**Fig. 1:**
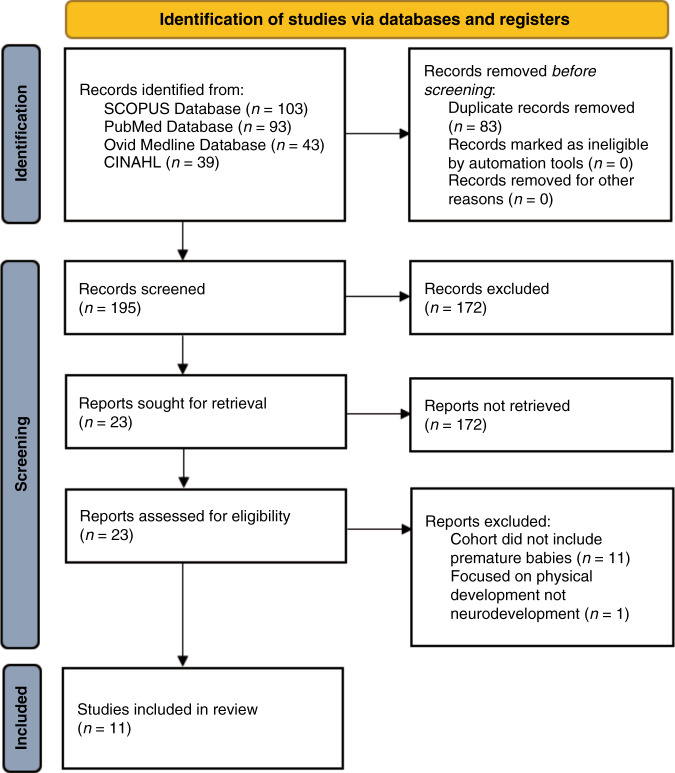
PRISMA flow diagram.

The literature search returned a collective 278 studies, with 195 remaining after duplicate removal. After screening titles and abstracts, 172 articles were excluded. The full texts of the remaining 23 articles were examined for eligibility. Eleven articles were excluded from the cohort, while one was excluded due to studying physical development rather than neurodevelopment. Thus, the literature search yielded 11 studies that were eligible for inclusion. Quality of these studies was assessed with the Newcastle–Ottawa Scale for cohort studies in Table 1. The included studies were conducted in 6 different countries: the United States of America, Canada, Japan, Scotland, England, and Australia. The process for identifying eligible articles is illustrated by the flow diagram in Fig. 1.

**Table 1:** Quality assessment of included studies in accordance with the Newcastle–Ottawa scale for cohort studies.

Study	Selection^a^	Comparability^b^	Outcome^c^
He et al. (2021)^16^	★★★★★	★	★★
Valavani et al. (2021)^18^	★★	★	★★
He et al. (2020)^17^	★★★★	★★	★★
Liu et al. (2020)^22^	★★	★	★★
Saha et al. (2020)^24^	★★★★	★	★★
Vassar et al. (2020)^19^	★★★★	★	★★★
Girault et al. (2019)^27^	★★★	★★	★★
Schadl et al. (2018)^20^	★★★★	★	★★
Kawahara et al. (2017)^21^	★★★	★	★★
Ball et al. (2016)^28^	★★★	★★	★★
Nishimura et al. (2016)^29^	★★★	★	★★

^a^Maximum 4 stars.

^b^Maximum 2 stars.

^c^Maximum 3 stars.

The included studies focused on two key topics: predicting and understanding neurodevelopmental outcomes. Eight papers focused on predicting neurodevelopmental outcomes, whilst also identifying the brain regions that contributed to the predictions made, as shown in Supplementary Table 1. The remaining three papers focused solely on understanding factors associated with neurodevelopmental outcomes, as shown in Supplementary Table 2.

Seven studies^16–22^ quantified neurodevelopment using the Bayley Scales of Infant Development Third Edition (Bailey-III).^23^ One study^24^ quantified neurodevelopment using the Neuro-Sensory Motor Developmental Assessment (NSMDA)^25^ while two used the Mullen Scales of Early Learning (MSEL).^26^

In terms of ML, the most popular technique was convolutional neural networks (CNN),^16,21,22,24^ with other techniques including fully connected neural networks (FCNN),^17,27^ random forest (RF),^18,28^ support vector machine (SVM),^28^ latent class growth analysis,^29^ logistic regression,^19,20,29^ and linear regression.^20^

In the following subsections, several key themes of the identified papers are discussed in depth.

### Classification of neurodevelopment as a binary outcome

Seven of the eight papers that sought to predict neurodevelopmental outcomes did so using a binary classification approach,^16–20,24,27^ where infants were identified as low or high risk of atypical neurodevelopment at an age between 18 and 24 months.

Each of these studies used Bayley-III, NSMDA, or MSEL scores to quantify neurodevelopment. The binary classification approach involved selecting a threshold score that split children into groups representing typical and atypical neurodevelopment.

Strategies for selecting a threshold varied throughout the literature. Of those studies that considered Bayley-III scores, most dichotomized the scores around a threshold of 85, or 1 standard deviation below the mean score.^17–20^ As Table 2 illustrates, language score was considered by three of these studies. The highest accuracy for language score was achieved by the RF model presented by Valavani.^18^ The logistic regression model by Schadl et al.^20^ presents accuracies of 100% and 88% for identifying cognitive and motor developmental delays, respectively; however, this was based on 8 participants. The study by He et al.^17^ also considered cognitive scores, achieving 81.5% accuracy and 0.86 area under the receiver-operator curve (AUROC), indicating that their CNN model has a good ability to distinguish between low and high-risk infants.

**Table 2:** Comparison of studies that aimed to classify infants as high or low risk based on a threshold of 85 using the Bayley-III scales.

Study	Balanced accuracy	AUROC
Valavani et al. (2021)^18^	91% (language)	Not provided
He et al. (2020)^17^	81.5% (cognitive), 68.9% (language), 73.9% (motor)	0.86 (cognitive), 0.66 (language), 0.84 (motor)
Vassar et al. (2020)^19^	58% (language composite), 66% (expressive language), 48.5% (receptive language)	0.502 (language composite), 0.617 (expressive language), 0.322 (receptive language)
Schadl et al. (2018)^20^	100% (cognitive), 88% (motor)	1 (cognitive), 0.912 (motor)

He et al.^16^ also used Bayley-III scores to assess neurodevelopment, dichotomizing outcomes around a threshold of 90. Their CNN model performed well in predicting cognitive, language, and motor deficits, achieving accuracies of 88.4%, 87.2%, and 86.7%, respectively. AUROC was also strong at 0.87, 0.85, and 0.85, respectively, indicating that the model is skilled at distinguishing between low and high risk.

Two studies classified risk groups based on alternative scales. Saha et al.^24^ assessed motor development with the NMSDA scale. Their model aimed to predict whether an infant would exhibit normal motor development or abnormal motor development at 2 years old, achieving an accuracy of 73% and AUROC of 0.72. Meanwhile, Girault et al.^27^ used the composite MSEL score to assess neurodevelopment, grouping outcomes into above median (score < 110) and below median (score > 110) groups. Their model then aimed to predict a preterm infant’s neurodevelopment at age 2, achieving accuracy of 83.8%.

### Predicting neurodevelopment as a value on a continuous scale

Three studies^16,21,27^ aimed to predict neurodevelopmental scores on a scale. Kawahara et al.^21^ presented a CNN model that aimed to predict Bayley-III scores at 18 months, with scores standardized between 50 and 155. Their strongest model achieved mean absolute error (MAE) values of 10.640 and 10.493 for motor and cognitive outcomes, respectively; however, correlation between true and predicted values was very low.

He et al.^16^ sought to predict Bayley-III scores at age 2 years with their CNN model. They standardized Bayley-III scores between 40 and 160. Their model achieved MAEs of 11.7, 10.5, and 11.6 in predicting cognitive, language, and motor scores, respectively. Correlation between true and predicted values ranged from 0.62 to 0.63.

Lastly, the deep FCNN model presented by Girault et al.^27^ aimed to predict MSEL cognitive scores scaled between 49 and 155. This study achieved an MAE of 4.47 and a strong correlation of *r* = 0.956 when predicting neurodevelopmental outcomes at the age of 2 years.

### Identification of features associated with neurodevelopmental outcomes

All studies sought to identify brain regions, connections, and other features predictive of neurodevelopmental outcomes. Several studies also considered clinical and demographic variables.

Schadl et al.^20^ used linear regression models to identify WM microstructures features obtained via diffusion tensor imaging (DTI) that correlated strongly with neurodevelopmental outcomes. Their findings showed that cognitive impairment was highly correlated with mean diffusivity (MD) of the right middle-temporal gyrus, right cingulate-cingulum, and left caudate in infants scanned at near-term corrected age. Meanwhile, motor impairment was linked with fractional anisotropy (FA) of the left precuneus and right hippocampus, and MD of the right superior occipital gyrus.

A linear regression approach was also used by Vassar et al.,^19^ whose study found that the severity of WM abnormality derived from magnetic resonance imagery (MRI) images was predictive of negative language outcomes. In addition, features obtained via DTI including MD of the right sagittal stratum and right inferior occipital gyrus along with the axial diffusivity (AD) of the right lingual gyrus were identified as strongly correlated with overall language outcomes.

Linear regression was used by Liu et al.^22^ to correlate relative brain age (RBA) of various regions of interest with neurodevelopmental outcomes. Their findings suggested that the RBAs of the left hemisphere precentral, superior frontal, inferior orbitofrontal, insular, middle cingulate, and posterior cingulate cortices were correlated with cognitive development at age 3 years. RBAs for the left hemisphere anterior cingulate and superior temporal pole cortices were shown to correlate with language outcomes, while no region was found to strongly correlate with motor outcomes.

Logistic regression was utilized by Nishimura et al.^29^ to identify clinical variables that contributed to delayed development. Their findings indicate that significant delay is linked with male sex and small-for-gestational-age birth.

Class activation mapping was used by Saha et al.^24^ to identify regions of FA maps that were most predictive of atypical motor development. Their findings indicated that the motor cortex and somatosensory regions were strongly correlated with neurodevelopment, while the cerebellum, and occipital and frontal lobes were also correlated.

Girault et al.^27^ used a backtracking approach to find features that contributed strongly to the output of cognitive development. They identified that WM connections between the frontal lobe and other regions are correlated with outcomes.

Backtracking was also used by Valavani et al.^18^ Their findings suggest that language delay was correlated with features including peak width of skeletonized FA, radial diffusivity, and AD. Correlation was also found with male sex, being a twin, and incomplete or no antenatal corticosteroid treatment.

Backtracking was used by Ball et al.^28^ to identify functional brain connections that differed between full-term infants and preterm infants at a full-term equivalent age. Their findings identified substantial functional connectivity differences in the basal ganglia and frontal regions of the brain, with connections appearing stronger in full-term infants.

Kawahara et al.^21^ used a partial derivative method to determine WM connections linked with outcomes. Their findings suggest that many brain connections of the right middle frontal gyrus are correlated with motor and cognitive scores. The left precuneus, fusiform gyrus, superior frontal gyrus, and right lingual gyrus are also identified as key connection regions.

A partial derivative approach is also used by He et al.^17^ to identify regions associated with neurodevelopmental outcomes, finding that the thalamus, middle-temporal gyrus, and inferior frontal gyrus were the strongest predictors of neurodevelopment. Their study also considered clinical variables, identifying birth weight, gestational age, bronchopulmonary dysplasia, and retinopathy of prematurity as predictors of neurodevelopmental outcomes.

In a later study, He et al.^16^ again utilized a partial derivative approach to assess a broader range of brain features and clinical variables. Their findings suggested that several functional connections were highly correlated with neurodevelopmental outcomes, primarily interhemispheric connections involving frontal, limbic, occipital, temporal, and parietal lobes. Structural connections were also correlated with neurodevelopment, with the most important connections found in the right hemisphere. In a comparison between diffuse WM abnormality (DWMA), functional connectivity, structural connectivity, and clinical variables, it was found that DWMA was the most correlated with outcomes. However, the strongest predictive performance was achieved by the model that used all four feature types as inputs, suggesting that all offer information regarding neurodevelopmental trajectory.

### Neural networks for predicting and understanding neurodevelopmental outcomes

Six of the studies identified by this paper utilized neural networks (NNs) for predicting neurodevelopmental outcomes, with four utilizing CNNs^16,21,24^ and two using FCNNs.^17,27^

He et al.^16^ used a deep CNN architecture to interpret functional and structural connectomes alongside clinical variables and WM abnormalities to predict Bayley-III scores. Kawahara et al.^21^ used a shallower CNN architecture to process structural connectomes and generate Bayley-III score predictions. Across both studies, low MAE values were achieved—ranging between 10.5 and 11.7 for different Bayley-III scores.

CNN architectures were also used by Saha et al.^24^ to interpret FA images of WM structures and classify infants into binary risk groups for motor developmental delay based on NSMDA scoring. CNN again performed strongly at this task, achieving an accuracy of 73%.

One study^22^ sought to use a graph-based CNN to predict the apparent age of the brain irrespective of the actual infant’s age. In this case, cortical surfaces of the brain were represented as a two-dimensional graph and used as an input to the CNN. The CNN then predicted RBA, which was thereafter shown to strongly correlate with neurodevelopment at age 3 years. Two studies focused on FCNN, traditional NNs where every neuron in one layer is connected to every neuron of the next layer. FCNNs are computationally expensive and risk overfitting to the training data, thus preventing accurate prediction on testing data. However, FCNNs have the advantage of viewing all information, rather than reducing feature dimensionality as with CNN. Both studies that utilized FCNNs sought to interpret connectomes.^17,27^

He et al.^17^ utilized FCNN to process functional and structural connectomes alongside clinical variables to classify infants into binary risk groups, achieving accuracies of 68.9% and 81.5% for language and cognitive development risk, respectively. This is markedly lower than their later study^16^ that utilized CNN.

Girault et al.^27^ implemented a deep FCNN structure to predict MSEL scores and binary risk classes based on the WM connectome. The MAE achieved in this study was very low at 4.47, with a strong correlation between true and predicted values of *r* = 0.956.

The study by Kawahara et al.^21^ compared their CNN model with several other models, including FCNNs. Their findings suggest that CNN outperforms FCNN in predicting Bayley-III scores from structural connectomes.

### Other machine learning techniques for predicting and understanding neurodevelopmental outcomes

Aside from NNs, techniques used in the literature included RF and logistic and linear regression. RF is an algorithm comprised of many decision trees that work together to predict outcomes. They are typically used on discrete features rather than image-based data. This is shown by Valavani et al.,^18^ who use RF to classify binary risk groups based on clinical, demographic, and brain structure variables. They achieve an accuracy of 91% in predicting language outcomes.

An RF-based strategy is also used to identify important features by Ball et al.,^28^ who then used these features to train a SVM. SVMs seek a plane of best fit between the input data and target outcome—in this case, the model aimed to classify functional connectome features as belonging to infants born either full-term or preterm.

Linear and logistic regression techniques are classical ML techniques. Linear regression focuses on predicting continuous outcomes like neurodevelopmental scale scores, while logistic regression is used for classification tasks. In the neonatal neurodevelopment literature, three studies used logistic regression^19,20,29^ and one used linear regression.^20^

Vassar et al.^19^ performed binary classification of risk groups, achieving a relatively low accuracy of 58% in predicting overall language development risk, while Schadl et al.^20^ achieved an accuracy of 100% in predicting motor development risk (albeit on a very small cohort of eight infants). Schadl et al.^18^ further investigated linear regression to identify features that showed the highest correlation with neurodevelopmental scores.

Lastly, Nishimura et al.^29^ used logistic regression to identify risk factors that were most predictive of neurodevelopmental delays. They further used latent class growth analysis to cluster retrospectively cluster children into groups of varying delay.

## Discussion

### Limitations in comparability

As illustrated in the section “Classification of neurodevelopment as a binary outcome”, there are inconsistencies in how atypical and typical neurodevelopment groups are identified. The most frequent approach in the literature is to use the Bayley-III scale with a threshold of 85. However, one study used an alternative threshold^16^ while others used different scales.^24,27^ As such, it becomes challenging to conduct a fair performance comparison between the methods used to classify the risk of atypical neurodevelopment.

Comparability remains an issue when contrasting the studies that aimed to predict a score on a neurodevelopment scale. The two studies that aimed to predict Bayley-III scores^16,21^ performed slightly different scaling of scores, meaning that the magnitude of MAE cannot be directly compared. The study that looked at predicting a neurodevelopment score^27^ looked at the MSEL score, preventing direct comparison to the other studies considered in this paper.

As the section “Identification of features associated with neurodevelopmental outcomes” demonstrated, the identification of features that were predictive of outcomes was approached in a wide variety of different manners. This is largely unavoidable given that different studies used highly dissimilar inputs and models; however, more consistency in approaches taken would help to improve comparability between studies in the future.

Another substantial issue that limits comparison between studies is the lack of an established database. One significant area for future research would be the development of a database that includes common medical images (MRI, fMRI, DTI, etc.) and clinical variables captured at or close to full-term equivalent age, along with neurodevelopmental outcomes at age 18–24 months. Such a database would be an invaluable resource, as it would support research that is comparable and reproducible.

### Binary risk classification compared to predicting neurodevelopmental scores

Two main approaches to prediction of neurodevelopment were identified in the “Results” section—binary risk classification and the prediction of a neurodevelopment score on a continuous scale. Of these two approaches, binary classification has the advantage of being the simplest to understand. Binary classification is also comparatively easy to implement and achieve strong performance, compared to predictions of a continuous scale. However, it provides little actionable information as no information is given about the likely severity of the neurodevelopmental outcomes in a child classified as at risk. It, therefore, remains a challenge for clinicians to determine the intervention required for the best long-term outcomes.

As such, we suggest that the models that aimed to predict neurodevelopmental scores on a continuous scale^16,20,21,27^ offer the most clinical value. A neurodevelopment score provides more information about the severity of the risk. This allows for appropriate intervention at an early age, enabling improved outcomes for each child. Initial studies show promising results, and thus this research direction is worth pursuing further.

An avenue not explored in the literature is multi-class classification. In this approach, infants could be classified into descriptive risk groups—for example, minimal/mild/moderate/significant/extreme risk. This would provide a metric that is descriptive and easier to understand than a raw number from a neurodevelopmental scale. Thus, this approach would better support treatment decisions than binary classification and would be more explainable to parents and non-experts than a neurodevelopmental score. This would be a worthwhile direction for future research.

Overall, the studies to date have shown that both binary risk classification and prediction of a score based on various neurodevelopmental scales are both feasible. Multi-class risk classification and neurodevelopmental score prediction are both recommended as directions for future research, as these better support clinical decision making.

### Brain features that are predictive of outcomes

In the section “Identification of features associated with neurodevelopmental outcomes”, it was shown that several studies identified WM structures as predictive of neurodevelopmental outcomes.^16–21,27^ In particular, WM abnormality was identified as highly predictive by two studies.^16,19^ The relationship between WM structures and neurodevelopmental outcomes has been previously established in the literature,^30^ and this is further confirmed by the studies identified in this review. Therefore, it is highly recommended that features quantifying WM structure and abnormalities continue to be included in future research.

Several studies also identified areas of interest that are not broadly acknowledged by prior literature. Structural connections, particularly those involving the frontal lobe, were identified as highly predictive of cognitive outcomes in two studies.^16,27^ However, one study identified predominantly interhemispheric connections^27^ while the other identified primarily connections within the right hemispheric, so disagreement remains about which connections are important. The frontal lobe was also identified as a region of interest in four studies;^17,21,22,24^ however, there was little consensus on other regions of importance.

Overall, results varied greatly in identifying features predictive of neurodevelopmental outcomes. It would be beneficial for future research to use comparable features to the studies to date, to move toward consensus on which brain regions and connections are correlated with neurodevelopment. Future research should consider structural connectivity, functional connectivity, WM abnormality, and clinical parameters when developing models, as all have been shown to carry information about neurodevelopmental trajectories.^16^

### Comparison of machine learning techniques

As observed in the section “Neural networks for predicting and understanding neurodevelopmental outcomes”, CNN and FCNN architectures have each been used in various studies looking to use structural and functional connectomes to predict neurodevelopmental outcomes,^16,17,21,27^ while CNN has also been used to assess outcomes from other image formats.^22,24^ Each of these studies demonstrated that NNs performed strongly at their respective tasks.

The key disadvantage of NNs is complexity. A typical NN contains hundreds of neurons. Contrasted with linear and logistic regressors, which could be represented as a single neuron, it is clear that NNs are less interpretable and more computationally expensive than their simpler predecessors.

Despite this, NNs have a key advantage in that they can make more complex connections between features and identify non-linear relationships; they can do no worse than a simple regressor. High computational cost is a decreasing problem as computers continue to become more powerful. To improve explainability of NNs, methods such as Shapley additive explanation scores^31^ and class activation mapping^32^ have been developed to illustrate how a network made its decisions.

The benefits of NNs outweigh the disadvantages when it comes to complex problems such as interpreting brain maps. As such, it is recommended that regressors are best suited to use as a benchmark. Other ML models, including NNs, can then be trained and tested with the goal of exceeding that benchmark.

It is also worth noting that several candidate ML approaches have not been explored extensively in the literature. Only one study has sought to predict neurodevelopmental outcomes with RF,^18^ with promising results. Further research into RF is needed to validate whether this technique is appropriate.

In terms of NNs, there are many architectures that remain unexplored. Future research could seek to develop novel models or investigate the use of pre-trained networks such as AlexNet^33^ and GoogleNet^34^ with fine-tuning to suit the neurodevelopmental prediction problem. This approach has been used in other medical imagery research domains, such as identifying glaucoma from optometry images^35^ and COVID-19 from x-rays.^36^

Interestingly, no study to date has examined residual NNs (ResNets),^37^ which are a variation on CNN networks that typically generalize to the data better than their predecessor. ResNet^37^ was first proposed in 2016 and has been widely used in image processing tasks such as diagnosing pneumonia from x-ray^38^ and Alzheimer’s disease from MRI.^39^

Overall, this field is young and there are many future research directions in terms of ML algorithms. There is a wide range of new and existing architectures that could be explored, and many pre-trained models that could be fine-tuned for neurodevelopment prediction and interpretation tasks.

## Conclusion

Early identification and intervention for children who are at risk of developmental disorders is critical to their well-being. The use of ML for predicting neurodevelopmental outcomes in preterm infants is a promising strategy for improving long-term outcomes. However, this remains a relatively new field with many future research opportunities.

The first key research question in this field is identifying how to classify outcomes. The majority of studies to date have aimed to predict whether preterm infants will develop typically or atypically, with some studies instead predicting an exact score on a neurodevelopmental scale. The latter option is preferable as it provides more actionable information for clinicians, allowing for tailored intervention and thus improving outcomes.

The next research questions lie in identifying suitable input features and suitable ML models. Studies aiming to interpret image-based information, such as structural and functional connectomes, tended toward CNNs and FCNNs. Meanwhile, studies that considered clinical variables or discrete brain features tended toward RF and regression approaches. As this review has identified that functional and structural characteristics of the brain are critical to strong predictive performance, it is suggested that NN strategies aimed at image processing are highly suitable for this task. It is further recommended that future studies consider novel CNN-based architectures, such as ResNet,^37^ in their research.

The most significant limitation for this field of research is the absence of a comprehensive and accessible database. The development of a database containing a wide range of imagery such as MRI, fMRI, DTI, and connectomes alongside clinical and demographic variables would be a significant and highly valuable contribution to the literature and to this field.

Overall, initial studies aiming to predict and understand neurodevelopmental trajectories in preterm infants with ML have shown promising results and interesting findings. However, much work remains to be done in order to find a consensus on ML strategies and conclusively identify features that are key to predicting neurodevelopmental delay. This field is likely to develop rapidly in years to come and offers many opportunities for future researchers.

## Supplementary information

Table S1

Table S2
