# Supplementary material for: Machine learning for understanding and predicting neurodevelopmental outcomes in premature infants: a systematic review
Source: Pediatr Res. 2022 May 31;93(2):293–9. doi: 10.1038/s41390-022-02120-w (PMC9153218; doi:10.1038/s41390-022-02120-w)
Supplement: Supplementary file 1 — Table S1 [file 41390_2022_2120_MOESM1_ESM.doc]

| **Study** | **Cohort** | **ML Technique** | **Input Features** | **Outcome** |
| --- | --- | --- | --- | --- |
| He et al (2021)^16^ | 261 preterm infants (training), 108 preterm infants (testing) | CNN | Functional connectome (from fMRI), structural connectome (from DTI), clinical variables, diffuse white matter abnormality quantifications (from MRI) | Bayley-III cognitive, language, and motor scores at 2 years, classification of low/high risk of cognitive, language, or motor delay at 2 years as defined by Bayley-III scoring, identification of brain regions associated with outcome |
| Valavani et al (2021)^18^ | 89 preterm infants | Random forest | Clinical variables, demographic variables, brain structure features (from dMRI) | Classification of low/high risk of language delay at 2 years old as defined by  Bayley-III scoring, identification of brain regions associated with outcome |
| He et al (2020)^17^ | 883 adults and children (pre-training), 291 infants  (training), 33 preterm infants (testing) | FCNN | Functional and structural connectomes (from MRI and fMRI), clinical variables | Classification of low/high risk of cognitive, language, or motor delay at 2 years old as defined by Bayley-III scoring, identification of brain regions associated with outcome |
| Saha et al (2020)^24^ | 77 preterm infants | CNN | Fractional anisotropy images (divided into smaller segments) | Classification of low/high risk of abnormal motor development at 2 years as defined by NSMDA scoring, identification of brain regions associated with outcome |
| Vassar et al (2020)^19^ | 92 very-low-birth-weight preterm infants | Multivariate logistic regression | White matter abnormality quantifications, cerebellar abnormality quantifications, white matter microstructure (from DTI) | Classification of low/high risk of language delay at 18-22 months as defined by Bayley-III scoring, identification of brain regions associated with outcome |
| Girault et al (2019)^27^ | 75 full-term infants (initial training and testing), 37 preterm infants (testing) | Deep FCNN | White matter structural connectome (from MRI) | Mullen Scales of Early Learning cognitive score at age 2 years, classification of low/high risk of cognitive and motor delay at age 2 years based on the Mullen Scales of Early Learning, identification of brain regions associated with outcomes |
| Schadl et al (2018)^20^ | 59 very-low-birth-weight preterm infants | Multivariate linear regression, logistic regression | Average fractional anisotropy, mean diffusivity, radial diffusivity and axial diffusivity calculated for 126 white matter regions (from DTI) | Classification of low/high risk of cognitive and motor delay at 18-22 months as defined by Bayley-III scoring (logistic regression), identification of brain regions associated with outcomes (linear regression) |
| Kawahara et al (2017)^21^ | 168 preterm infants | CNN | White matter connectomes (from DTI) | Bayley-III cognitive and motor scores at 18 months, identification of brain regions associated with outcomes. |

**Table S1 –** A comparison of machine learning approaches for predicting neurodevelopmental outcomes in preterm infants
