# Supplementary material for: Machine learning for understanding and predicting neurodevelopmental outcomes in premature infants: a systematic review
Source: Pediatr Res. 2022 May 31;93(2):293–9. doi: 10.1038/s41390-022-02120-w (PMC9153218; doi:10.1038/s41390-022-02120-w)
Supplement: Supplementary file 2 — Table S2 [file 41390_2022_2120_MOESM2_ESM.doc]

| **Study** | **Cohort** | **ML Technique** | **Input Features** | **Outcome** |
| --- | --- | --- | --- | --- |
| Liu et al (2020)^22^ | 138 preterm infants | Graph-based CNN | Cortical surface derived  from MRI scans | Prediction of brain age (from MRI), which  is then shown to correlate with cognitive, language and motor development at age 3 years as defined by Bayley-III scores |
| Ball et al (2016)^28^ | 109 preterm infants, 25 full-term infants | RF for feature selection, SVM for prediction | Features of functional connectome (from fMRI) as selected by RF | Identification of brain regions where substantial functionality differences exist between preterm infants at term corrected age and full-term infants, classification of preterm vs full-term infant based on connectome at full-term equivalent |
| Nishimura et al (2016)^29^ | 56 preterm infants, 896  full-term infants | Latent class growth analysis, multinomial logistic regression | Parental demographics, perinatal variables | Identification of risk factors for delayed development of gross motor, fine motor, visual reception, expressive language, and receptive language skills aged 1-24 months as defined by Mullen Scales of Early Learning |

**Table S2 –** A comparison of machine learning approaches for understanding contributing factors for neurodevelopmental outcomes in preterm infants
